# Prevention of Morbidity in sickle cell disease - qualitative outcomes, pain and quality of life in a randomised cross-over pilot trial of overnight supplementary oxygen and auto-adjusting continuous positive airways pressure (POMS2a): study protocol for a randomised controlled trial

**DOI:** 10.1186/s13063-015-0883-y

**Published:** 2015-08-25

**Authors:** Jo Howard, Baba Inusa, Christina Liossi, Eufemia Jacob, Patrick B Murphy, Nicholas Hart, Johanna Gavlak, Sati Sahota, Maria Chorozoglou, Carol Nwosu, Maureen Gwam, Atul Gupta, David C Rees, Swee Lay Thein, Isabel C Reading, Fenella J Kirkham, Man Yeung Edith Cheng

**Affiliations:** 1 Department of Haematology, Guy’s and St Thomas’ Hospitals NHS Foundation Trust, London, UK; 2 Evelina Children’s Hospital, Guy’s and St Thomas’ Hospitals NHS Foundation Trust, London, UK; 3 grid.5491.90000000419369297University of Southampton, Southampton, UK; 4 grid.19006.3e0000000096326718University of California Los Angeles, Los Angeles, CA USA; 5 grid.13097.3c0000000123226764King’s College London, London, UK; 6 Lane Fox Respiratory Unit, Guy’s and St Thomas’ Hospitals NHS Foundation Trust, London, UK; 7 grid.123047.30000000103590315Department of Child Health, University Hospital Southampton, Southampton, UK; 8 grid.83440.3b0000000121901201Neurosciences Unit, University College London Institute of Child Health, London, UK; 9 Sickle Cell and Young Stroke Survivors Charity, London, UK; 10 grid.46699.340000000403919020King’s College hospital, London, UK; 11 grid.123047.30000000103590315Research Design Service, University Hospital Southampton, Southampton, UK

**Keywords:** Sickle cell anaemia, Inherited diseases, Haemoglobin, Qualitative method, Statistical method, Randomised controlled trial

## Abstract

**Background:**

Sickle cell anaemia (SCA) is an inherited disorder of haemoglobin. Patients experience long**-**term health care problems, affecting quality of life (QOL) including frequent acute pain, which is difficult to document in trials except as hospital admissions. Pilot data suggests that overnight respiratory support, either supplementary oxygen or auto**-**adjusting continuous positive airways pressure (APAP), is safe and may have clinical benefit. This pilot trial aims to determine which intervention is more acceptable to participants and whether there are other advantages of one over the other, e.g. in respiratory function or haematological parameters, before conducting the Phase 2 trial of overnight respiratory support funded by the National Institutes of Health Research.

**Methods/Design:**

This is a pilot cross**-**over interventional trial with the order of interventions decided by simple randomization. Ten adults (age over 18 years) and 10 children (aged between 8 and 18 years) with homozygous sickle cell disease (haemoglobin SS, HbSS), recruited regardless of symptoms of sleep**-**disordered breathing, will undergo overnight pulse oximetry and will have two interventions, overnight oxygen and APAP, for a week each in randomised order with a washout week between interventions. Participants will complete online diaries via an iPad throughout the 29 days of the study and will complete QOL questionnaires and have measurement of haematology, biochemistry, spirometry and lung volumes (adults only) at 3 time points, at baseline and after each intervention, as well as in-depth semi-structured qualitative interviews after each intervention, carried out by an experienced psychologist. Both qualitative and statistical methods will be used to analyze the data. The primary outcome is qualitative data looking at participant experience from the transcribed interviews after each intervention. The participant’s view on feasibility, acceptability and preference will specifically be explored. The QOL, laboratory and lung function data will be compared with baseline for each arm.

**Discussion:**

Patient and public involvement is an integral part of this trial and the key outcome is the qualitative result, which is dependent on obtaining good quality data to advise on participant feasibility, acceptability and preference. This is being addressed by using a standard interview. The development of a pain endpoint is another important outcome and collecting daily measurements is likely to be challenging. Research results will be used to inform design of the Phase 2 trial.

**Trial registration:**

ISRCTN46078697 18 July 2014

**Electronic supplementary material:**

The online version of this article (doi:10.1186/s13063-015-0883-y) contains supplementary material, which is available to authorized users.

## Background

Sickle Cell Anaemia (SCA) is a recessively inherited disorder of haemoglobin, the protein which carries oxygen inside red blood cells. SCA affects an estimated 15,000 people [1] in the UK and patients experience long**-**term health care problems, including pain and neurocognitive problems, which affect quality of life. Emergency presentations are typically due to acute sickle cell pain [2, 3] and account for over 6000 emergency admissions and over 25,000 bed days per year [4]. Mortality in children in England has improved over recent years with around 99 % now surviving to 18 years [5]. Life expectancy is, however, shortened to 40–50 years [6, 7] and quality of life is compromised by chronic complications [8–16].

The prevalences of *intermittent* nocturnal haemoglobin oxygen desaturation, secondary to sleep**-**disordered breathing, and *sustained* daytime and nocturnal haemoglobin oxygen desaturation, are high in patients with SCA [17–22]. There is an association between low oxygen saturation (SpO_2_) and SCA complications, including stroke [12, 23], enuresis [14] and priapism [15] as well as painful crisis [3], although the latter is controversial [18], perhaps related to differences in documentation of painful crisis. Cognitive function, including impaired attention, is a particular problem in SCA [24–29], and may be linked to sleep**-**disordered breathing and oxygen desaturation [25–29], as it is in the general population.

Treatment options for sleep**-**disordered breathing and nocturnal hypoxia include continuous overnight oxygen via a concentrator and continuous positive airways pressure (CPAP) but there are few data on their use in SCA. These two interventions commonly used to reduce overnight hypoxic exposure have different modes of action and there are some preliminary data available for each:

1. Positive Airways Pressure. CPAP therapy reduces sleepiness in adults and children with obstructive sleep**-**disordered breathing in the general population and may improve cognition and intermediate vascular endpoints [30–33] but adherence is an issue [31]. Auto**-**adjustable CPAP (APAP) is more comfortable as the pressure support is only triggered when the obstruction occurs [34].
*Positive Airways Pressure in SCA* Positive airways pressure has been used short**-**term in SCA in acute chest crisis [35] and to prevent peri**-**operative complications [36]. In unselected children with SCA in our 6**-**week proof**-**of**-**concept randomised controlled trial (RCT), overnight respiratory support with APAP was safe and feasible, with excellent adherence in all 12 participants in the treatment arm and no suppression of erythropoiesis [37]. Improvement in cancellation (Wechsler Intelligence Scale for Children (WISC**-** ^IV^UK)), a measure of attention as well as processing speed, was seen in those on APAP compared with those not treated [37]. Pain frequency, defined as the number of days that pain was experienced in a 2**-**week period, improved in the treatment arm (*p* = 0.07) but this did not reach statistical significance, perhaps related to reduced statistical power due to reluctance to complete paper pain diaries (full data was only available for 8 of the 12 participants in each arm) [37]. As pain is the cardinal symptom in sickle cell disease, and is, therefore, an important endpoint in clinical trials, ensuring that all participants complete any diaries is important. With smartphone technology [38], daily pain intensity and site may be explored in addition to frequency [39], number of days in hospital [3] or number of admissions [20].
2. Overnight oxygen is well**-**established for the treatment of hypoxia secondary to lung diseases, such as chronic obstructive airways disease in adults or bronchopulmonary dysplasia. Uncontrolled hypercapnia is a risk in settings where respiratory failure may occur [40].
*Oxygen supplementation in SCA* There are few data on the safety of oxygen administration in people with SCA and most data is available over short periods of time, typically in the management of acute crisis. The two main concerns in using overnight oxygen in SCA are the suppression of erythropoiesis and rebound pain, which has been documented with the administration of high flow rates of oxygen throughout 24**-**hour periods for several days [41]. However, reticulocytosis was documented in a child whose abdominal pain was relieved by 18 days placement within an oxygen tent [42]. In addition, erythropoietin levels did not fall in non**-**hypoxic adults randomised to receive oxygen during a painful crisis [43]. Although the duration of painful crisis was not reduced by the administration of oxygen in this study [43] or a paediatric trial designed to administer 50 % oxygen [44, 45], there was no evidence of rebound pain.
  One report audited the use of long**-**term oxygen supplementation in SCA. Ip et al [46] described 6 adults with SCA (age range 20–45 years; 4 women), who had been commenced on oxygen therapy (1**–**2 l/minute) in the previous 2 years because of nocturnal hypoxia, defined as oxygen saturations < 90 % for > 30 % of the night. A detailed case notes review showed a mean increase in haemoglobin and reticulocyte count, with no change in erythropoietin and painful episodes.

There is a possibility that overnight respiratory support improves daytime lung function [47, 48] and oxygen saturation. Both obstructive and restrictive lung disease have been reported in patients with SCA [49] and it is possible that there are physiological advantages for overnight oxygen or APAP, e.g. in improving daytime oxygen saturation [37] through improving gas exchange by overcoming upper or lower airway obstruction or increasing lung volume.

In addition to requiring additional safety data and exploring the physiological effects of overnight respiratory support, further work is needed to determine whether one of these alternatives is preferable to patients in terms of the inconvenience when compared with any possible benefits. To attempt to improve completion of diary data, the feasibility and acceptability of daily data collection on presence, site and severity of pain using a visual analogue scale for highest, pain and lowest daily pain using smartphone technology on an iPad mini (Apple Inc., Cupertino, CA, USA) also requires assessment.

The National Institute of Health Research (NIHR) Research for Participant Benefit (RfPB) stream has funded our group to undertake a Phase 2 randomised 2**-**arm trial of overnight respiratory support or standard treatment. As there are very few pilot data involving treatment for sleep**-**disordered breathing in this condition, it is important to assess the acceptability of overnight oxygen supplementation compared with APAP in participants before deciding on which form of overnight respiratory support to use as the treatment arm in the Phase 2 trial. In the participants and public involvement (PPI) work for the RfPB submission, no participant preference came out between the proposed two interventions to help make a decision on which intervention to choose, but these participants did not have first**-**hand experience of using either device. This pilot phase is designed to examine patient preferences after 1 week of using each device in randomised order and will also determine whether there is evidence that either form of overnight respiratory support has a short**-**term beneficial or detrimental effect on haematological variables or lung function.

### Aims and objectives

The aim of this pilot study (Prevention of Morbidity in SCA, POMS2a; Table 1) is to ascertain which intervention (overnight oxygen or APAP) is more acceptable to participants by asking them to use both interventions for 1 week each, with each intervention followed by an in depth semi**-**structured qualitative interview. It will also assess the quantitative methodologies, which are to be used in the next larger trial (POMS2b, the Phase 2 trial).

**Table 1:** Protocol details

Protocol title:	Prevention of Morbidity in sickle cell disease 2a – pilot phase (Improvement of Pain and Quality of Life in Participant with Sickle Cell Disease with Nocturnal Oxygen Therapy or Auto**-**adjusting Continuous Positive Airways Pressure: pilot phase)	
Protocol number and date	Protocol Version 8 5February 2014	
Protocol chair/principal investigator:	Fenella Kirkham MD FRCPCH	
	Professor of Paediatric Neurology	
	Institute of Child Health (University College London)	
	Office: 44 207**-**905**-**2968	
	Fax: 44 207**-**833**-**9469	
	Email: fenella.kirkham@ucl.ac.uk	
Protocol team/co**-**investigators:	Prof David Rees MD MRCP MRCPath	Dr Jo Howard MRCP FRCPath
	Professor in Paediatric Haematology	Consultant Haematologist
	King's College Hospital	Guy’s and St Thomas’ NHS Foundation Trust
	Tel: 0207 346 3242	Tel: 0207 188 2741
	Fax 0207 346 4689	Fax 0207 188 2728
	Email: david.rees@kingsch.nhs.uk	Email: jo.howard@gstt.nhs.uk
	Dr Baba Inusa MB FRCPCH	Prof Swee Lay Thein
	Consultant Paediatrician,	Professor of Molecular Haematology
	Evelina Children’s Hospital,	King’s College Hospital
	London SE1	Tel: 0207 848 5443
	Tel: 020 7177 7177	sl.thein@kcl.ac.uk
	Email: Baba.Inusa@gstt.nhs.uk	
	Dr Christina Liossi	Dr Nicholas Hart
	Senior Lecturer in Health Psychology	Consultant in Respiratory and Critical Care
	University of Southampton	Guy’s and St Thomas’ NHS
	Tel: 02380594645	Foundation Trust
	Fax	Tel: 0207 188 7608
	Email: cliossi@soton.ac.uk	Email: Nicholas.hart@gstt.nhs.uk
	Ms Carol Nwosu	Dr Man Ying Edith Cheng
	Chief Executive Officer	Statistician
	Sickle Cell and Young Stroke Survivors	University of Southampton
	Suite R, 7th Floor, Hannibal House	Research Design Service
	London UK	UK
	Tel : 0844209292	Tel: 02380795704
	Email: Carolnwosu@scyss.org	Email: m.y.cheng@soton.ac.uk
	Associate Professor	Ms Maria Chorozoglou
	Assistant Professor Paediatrics	Snr RF in Health Economics
	Department of Nursing	Wessex Institute
	University of California	Faculty of Medicine
	Tel: 310 267 1823	University of Southamptom
	Email: eufemia@sonnet.ucla.edu	UK
		Tel : 02380597457
		Email: M.Chorozoglou@soton.ac.uk
Study sites	Guy’s and St Thomas’ NHS Foundation Trust	

There are 4 objectives for this study: (1) to assess whether overnight oxygen therapy or APAP is more acceptable to participants, (2) to assess whether there are any physiological or clinical benefits or risks of overnight oxygen therapy or APAP, (3) to assess the feasibility of using smartphone technology to collect daily information on site and severity of pain and (4) to identify the main cost drivers and potential cost implications of providing the intervention.

## Methods/Design

The study was given approval by NRES Committee East of England – Cambridge South (14/EE/0163) on 3 June 2014. Local ethical permission was granted by Guy’s and St Thomas’ hospital NHS Foundation Trust. This is a pilot cross**-**over interventional trial. Participants will have 2 interventions, overnight oxygen and APAP, for a week each in randomised order (weeks 2 and 4). There will be a week of baseline data collection (week 1), and a week of washout between the interventions (week 3).

Randomisation will be done by simple randomisation by an independent statistician at the University of Southampton. It will not be possible to blind the participant, study co**-**ordinator, sleep physiologist or psychologist to the order of treatment. However, the principal investigator, statistician and technician performing spirometry, i.e. those responsible for documenting the quantitative endpoints, will be blinded to which intervention is given in which order.

### Qualitative evaluation

At the end of each intervention period a qualitative interview will be conducted by a psychologist. Participating children and young people aged between 8 and 18 years will be offered the choice of joint (children paired with their parents) or separate interviews. Parents will provide consent and children will be asked to assent in qualitative interviews. Interviews will be tape**-**recorded and fully transcribed. Inductive qualitative semi**-**structured interviews will be used to gain a rich, in**-**depth understanding of participants’ experiences and appraisals of oxygen therapy and APAP across age groups and participant status (i.e. patient versus carer).

### Participants

This pilot study will involve 20 participants, recruited regardless of symptoms of sleep**-**disordered breathing, which includes 10 children (age between 8 and 18 years) and 10 adults (age over 18 and above). All participants will be enrolled into the study for 29 days (i.e. 4 weeks).

Consenting participants with HbSS who are aged > 8 years and attend at Guy’s and St Thomas’ NHS Foundation Trust are eligible for this study. Participants will be excluded if they already have overnight respiratory support, if they have existing respiratory or decompensated cardiac failure or if they have any contra**-**indications to APAP therapy. Both inclusion and exclusion criteria are listed in Table 2.

**Table 2:** Participant eligibility criteria for participation in the POMS2a trial

Inclusion criteria	
1	Recruitment will be through sickle cell clinics at Guy’s and St Thomas’ (including Evelina Children’s Hospital)
2	Age > 8 years
3	Informed consent with assent in accordance with the institutional policies (UK ethical committee) and European or US Federal guidelines must be signed by the participant or participant's parent or legally authorised guardian acknowledging written consent to join the study. Where appropriate, participants < 16 years will be requested to give their assent to join the study
4	HbSS diagnosed by standard techniques (HPLC, IEF and MS). Participating institutions must submit documentation of the diagnostic haemoglobin analysis
5	Able to speak and understand English
6	Participant or parent/guardian able to use iPad mini via wireless
Exclusion criteria	
1	Participant already on overnight respiratory support, or has used it in the past
2	Hospital admission for acute sickle complication within the past 1 month
3	Participant with > 6 admissions for acute sickle complications within the past 12 months
4	Existing respiratory failure
5	Decompensated cardiac failure
6	History of severe epistaxis
7	Trans-sphenoidal surgery, or trauma that could have left a cranio-nasopharyngeal fistula
8	Perforated ear drum
9	Bullous lung disease
10	Bypassed upper airway
11	Pneumothorax
12	Participant at increased risk of aspiration
13	Pneumocephalus has been reported in a participant using nasal Continuous Positive Airways Pressure. Caution should be used when prescribing APAP for susceptible participants such as those with: cerebral spinal fluid (CSF) leaks, abnormalities of the cribriform plate, prior history of head trauma, and/or pneumocephalus
14	Pregnancy
15	Participants on chronic blood transfusion regimes, or has had blood transfusion within past 3 months
16	Any acute or chronic condition which would limit the participant’s ability to complete the study
Temporary exclusion criteria	
17	Sinus or middle ear infection

### Screening visit

Inclusion and exclusion criteria will be reviewed at the screening visit. After the participant (and parents for paediatric participants) have familiarised themselves with the trial protocol and have given written informed consent, relevant clinical history will be taken.

### Procedure (Table 3, Fig. 1)

**Table 3:** Details of study flow and duration of each interventions period

	Screening visit	Trial entry (Day 1)	Days 8–14	Days 15–21	Days 22–28	Day 29
Participant given PIS	x					
Inclusion/Exclusion reviewed	x	x				
Consent signed		x				
Daytime oximetry		x		x		x
Overnight oximeter issued		x				
Oximeter collected			x			
Spirometry		x		x		x
Lung volume testing (adults)		x		x		x
Quality of life evaluation		x		x		x
Blood tests/urine tests		x		x		x
Smartphone issued		x				
Daily pain diary for next week		x	x	x	x	
Intervention 1 commenced			x			
Sleep physiologist to review participant at home to set up intervention 1, review participant and adverse events			x			
Intervention 1 stopped (courier to pick up)				x		
Intervention 2 commenced					x	
Sleep physiologist to review participant at home to set up intervention 2, review participant and adverse events					x	
Intervention 2 stopped (courier to pick up)						x
Qualitative interview with participant and parent/guardian				x		x
Medical/nursing review of compliance and adverse event reporting				x		x

**Fig. 1:**
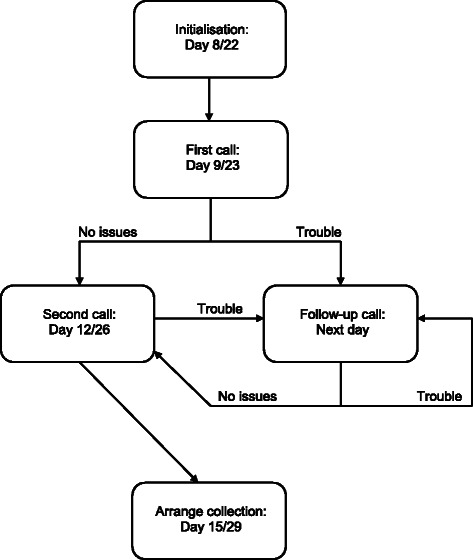
Flow chart for the interventions and support from the respiratory physiologist

On day 1, the patient will attend the Day Care Unit at the hospital and data including baseline blood tests, PEDS**-**QL quality of life [50], daytime oximetry, will be collected. Adults will undergo lung function (spirometry and lung volume). The participant will be issued with an iPad mini with a validated smartphone app [38] for rating pain and symptom assessment and will be asked to complete daily data collection using this device for the duration of the whole study, taking approximately 5 minutes per day. They will be given an overnight oximeter to take home and will be asked to use this for 2 nights over the next week. The randomization codes will be provided at the start of the study by an independent statistician from the University of Southampton, who will use simple randomization to generate the order.

On day 8, the participant will have the first intervention (intervention 1), either oxygen or APAP, which will be delivered to their home and set up by the respiratory physiologist, or if they prefer, they can take the treatment home after explanation by the respiratory physiologist. They will be asked to use this for 7 nights and will have planned support calls (Fig. 1). The overnight oximeter will be collected by the respiratory physiologist and the data analysed. On day 15 the participant will return to the hospital Day Care Unit for medical assessment, blood tests, PEDS**-**QL quality of life assessment, daytime oximetry and the first qualitative assessment with lung function studies for the adults. Days 16**–**21 will be a washout phase with no intervention given, but ongoing daily data collection via the smartphone app on the iPad mini. On day 22 the participant will have the other intervention (intervention 2) installed at home and they will be asked to use this for the next 7 nights. On day 29, the participant will go to the Day Care Unit at the hospital for medical assessment, blood tests, PEDS**-**QL quality of life assessment, daytime oximetry and lung function tests (adults) and the second qualitative assessment. Adverse events and serious adverse events will be reported to the sponsor (Tables 4 and 5).

**Table 4:** Laboratory findings to be reported to the sponsor within a maximum of 7 days

Variables	Measurement level that may be consider as adverse events
Haemoglobin	Fall of > 20 g/l from baseline is significant
Reticulocytes	10–100 x 10^9^ fall < 10 × 10^9^ is significant
Lactate dehydrogenase	Increase of > 1.5 x from baseline is significant.
Bilirubin	Increase of > 1.5 x from baseline is significant
Creatinine	Increase of > 1.5 from baseline is significant
Erythropoeitin	Fall of > 50 % from baseline is significant
Oximetry	Decrease in baseline oximetry of > 3 % from baseline

**Table 5:** The relationship between an adverse event and the intervention

Type of adverse events and relationship to the intervention	Description
Unrelated	• No temporal association to study intervention
	• An alternative aetiology has been established
	• The event does not follow the known pattern of response to study intervention
	• The event does not reappear or worsen with re-challenge
Unlikely to be related	• No temporal association to study intervention
	• Event could readily be produced by clinical state, environmental or other interventions
	• The event does not follow the known pattern of response to study intervention
	• The event does not reappear or worsen with re-challenge
Possibly related	• Reasonable temporal relationship to study intervention
	• The event is not readily produced by clinical state, environmental, or other interventions
	• The event follows a known pattern of response to the study intervention or as yet unknown pattern of response
Definitely related	• There is a reasonable temporal relationship to the study intervention
	• The event is not readily produced by clinical state, environmental, or other interventions
	• The event follows a known pattern of response to the study intervention
	• The event decreases with de-challenge and recurs with re-challenge
Unassessable	• Anything that does not fall into the above categories

The qualitative researcher will undertake interviews at the end of both interventions for all 20 participants and 10 parents/guardians to determine participant preference for one or other intervention. Participants will have a phone call at days 9 and 23 from the sleep physiologist to ensure they are using the equipment appropriately, and further phone support will be available if necessary. The sequence of the proposed investigations and the duration of the trial period is given in Table 3 and the participants’ journey through the interventions is shown in Fig. 1.

### Withdrawal criteria

Participants who wish to withdraw from the study are free to do so. If this occurs they will be asked if they would be willing for us to document withdrawal and the reasons. Participants will be withdrawn if they experience a serious adverse event and in this situation they will be asked if they will continue with qualitative assessment if appropriate. The trial intervention will be stopped if participants are admitted to hospital with an acute sickle complication. In this situation they will be asked if they will continue with the qualitative assessment and if they have only have received one intervention, if they would be happy to have the second intervention.

All adverse events will be documented for these participants as if they had remained in the trial. They will be asked to continue with qualitative assessments. Participants who withdraw may be replaced if appropriate participants are available and consent to the study. Participants will continue to be followed**-**up under the normal clinical care pathway at Guy’s and St Thomas’ NHS Foundation Trust. Participants who develop evidence of suppressed erythropoiesis will discontinue the trial. If they have not had a Parvovirus infection, the blind will be broken.

### Interventions

The two interventions in this study are overnight oxygen therapy and APAP. Both APAP and nocturnal oxygen therapy are non**-**invasive. Support from a respiratory physiologist with experience of APAP and nocturnal oxygen therapy will be available to maximise compliance with the interventions (Fig. 1). Details of each of the interventions are listed below:

### Intervention: APAP

The REMstar® Auto System (Philips Respironics, Chichester, UK) is an APAP device designed for the treatment of obstructive sleep**-**disordered breathing. When set in the APAP mode, the system will monitor breathing whilst sleeping and automatically adjust the pressure to overcome upper airway obstruction. APAP will be administered via a nasal or oral**-**nasal mask. APAP will be set at 4 cmH_2_O with an upper limit of 10 cmH_2_O.

### Intervention: oxygen therapy

Nocturnal oxygen therapy: oxygen concentrator device supplied by Philips Respironics (Chichester, UK). Oxygen therapy is administered via nasal cannula or mask depending on participant preference. Nocturnal oxygen therapy will be administered at 0.5 L/min, in children which was the level most commonly used in the previous trial [37] and 1 L/min in adults.

### Adherence to intervention

For the APAP arm, compliance and adherence to treatment will be formally assessed using specially designed software (Encore Pro™ data management software, Philips/Respironics) and a SmartCard that records both qualitative and quantitative data on a single mail**-**in card. This will be assessed at the end of the 7**-**day intervention period. There is no such capability for the oxygen concentrators and the participant/carer will be asked to record the hours of use. The sleep physiologist will phone participants at 24 hours after starting each intervention and mid treatment. They will provide further telephone support should study participants and their family have any questions/issues with the study treatment or if participants identify a problem (Fig. 1).

### Primary outcome

#### Measuring participant benefit

This is a qualitative study looking at participant experience from the transcribed interviews after each intervention as the primary outcome. The participant’s view on feasibility, acceptability and preference will specifically be explored. Established guidelines for thematic analysis will be followed [51] and augmented with charting procedures from framework analysis [52, 53]. First, one researcher will listen to, read and reread the interviews and transcripts. The interviews will be then coded line**-**by**-**line [53]. A coding manual will be created, and codes that appear most useful to the research question will be applied to the rest of the transcripts [53]. This analysis will be iterative, involving constant comparison and refinement between codes and transcripts to ensure that codes are being used consistently and reflect the data. Codes identifying similar aspects of the data will be clustered together under themes and subthemes. Having identified the main themes, participants will be grouped in a chart according to intervention and participant status, and their talk that relates to each of the themes will be summarised (based on the charting techniques described in framework analysis [52, 53].

### Secondary endpoints

#### Pain

Using the smartphone technology [38] which downloads automatically, information on (a) pain characteristics (intensity, location, quality), (b) pain medications and non**-**pharmacological strategies used for pain, (c) health care visits will be collected in the pilot to test out the methodology and to determine whether there is any obvious effect of either intervention or its withdrawal, particularly if detrimental. This is important as the first outcome measure for trial 2b will be average pain intensity during the 2 observation periods.

#### Adverse events

The Clinical Report Forms (CRFs) for reporting adverse events will be trialled during this pilot to show efficacy in recording and reporting adverse events.

Daytime oxygen saturation will be collected before and after each intervention to determine whether there is any obvious effect of either intervention or its withdrawal, particularly if detrimental.

#### Lung function

Spirometry and lung volume will be collected before and after each intervention to determine whether there is any obvious effect of either intervention or its withdrawal, particularly if detrimental.

#### Quality of life data

Age appropriate versions (paediatric or adult) of the PEDS**-**QL quality of life measure [50], including the sickle module, will be collected at the beginning of trial and after each intervention to determine whether there is any effect of either intervention.

### Safety measurement

Safety assessment will be undertaken by the chief investigator (FJK) liaising with the local adult (JH) and paediatric (BI) principal investigators at the local sites by (1) review of adverse events (including pain diary) at days 15 and 29**,** (2) review of basic haematological and biochemical parameters at days 1, 15 and 29. This will include full blood count, reticulocyte count, lactate dehydrogenase (LDH), bilirubin, creatinine, erythropoietin, albumin creatinine ratio and (3) daytime oximetry at days 1, 15 and 29. The value(s) or range(s) for medical, laboratory and/or technical procedure(s) are given in Table 6.

**Table 6:** Normal measurement range

Parameters	Normal measurement and range
Pain diary	Pain rates whilst receiving the intervention will be reviewed with baseline pain rates
Haemoglobin	130–170 g/l normal range in men, 120–150 g/l in women, 115–145 g/l in children
Haemoglobin F %	0–1.5 %
Reticulocytes	10–100 x 10^9^
Lactate dehydrogenase	24–280 IU/l
Bilirubin	0–21 μmol/l
Creatinine	45–85 μmol/l
Erythropoietin	5–25 IU/l
Daytime oximetry	>94 %

### Statistical analysis

The preliminary data on participant preference and physiology will be analysed at the end of this pilot phase and will be used to make a final decision about which intervention should be used for trial 2b (the Phase 2 study). This is a pilot phase; all statistical analyses will be treated as preliminary and exploratory and will mainly be descriptive [54]. We will investigate factors that influence recruitment rate, acceptability, adherence and loss to follow**-**up. The variability of outcome measures will be reported and any indication of improvement on any of the treatment will be explored informally: e.g. daily pain rate from the smartphone app, admissions to hospital for complications and laboratory parameters. The decision will be made primarily according to participant preference and, if this is at equipoise, on any apparent physiological benefits, e.g. higher daytime oxygen saturations, improved lung function or rates of adverse events. The cost implications will be assessed and relative long**-**term costs to the NHS will be taken into account if there is still equipoise.

The study will be reported in accordance with the CONSORT (Consolidated Standards of Reporting Trials) 2010 statement (Additional file 1) (or latest version if it is available at the time of reporting) [55]. A baseline table will be included to compare important demographic and clinical characteristics between those who started intervention 1 first and those who started intervention 2 first. In total there 3 time points, baseline, after intervention 1 and after intervention 2; all variables will be reported at all 3 time points. Any deviations from the original statistical plan will be incorporated, with full explanation, into a revised version of the protocol.

## Discussion

This is a pilot cross**-**over interventional trial involving children and adults with SCA. Patient and public involvement has been an integral part of this trial and the key outcome is qualitative and dependent on obtaining good quality data to advise on participant feasibility, acceptability and preference. This is being addressed by using a standard interview, which is carried out by a designated psychologist. The development of a pain endpoint is another important outcome and collecting daily measurements is likely to be challenging. This has been addressed by using smartphone technology via an iPad mini, which can connect to wireless networks but does not require a phone contract [38], which is cost**-**effective and should be easier for participants to complete. Assessment of the usefulness of this technology is important as the first outcome measure for the Phase 2 trial, POMS2b, will be average pain intensity during the 2 observation periods and we plan to use the same technology if this proves successful in the pilot. For this pilot study, we are including patients regardless of pain frequency and severity, although it is possible that patients with a significant burden of pain might have a different spread of preferences. In the Phase 2 trial there is a case for including only patients with chronic pain, screened with an appropriate questionnaire for burden of pain [56]. The patient’s experiences and acceptability of both interventions, and statistical evaluation of pain, adverse events, safety data and physiological data will all be taken into account to determine the most acceptable intervention. The most acceptable intervention will be used in the second phase of the larger proof**-**of**-**concept study (POMS2b).

A potential risk is that the participants and their families do not feel adequately supported to agree to recruitment or to continue in their allocated intervention. The sleep physiologist (who will not be blinded to the interventions) will provide easily accessible advice and support. She will routinely call the patient the day after the intervention has started but will also be available to answer additional question during the time of the intervention. Recruitment may also be a challenge but experienced clinical haematologists are co**-**applicants in order to maximise the chance of steadily recruiting participants who will commit to the study and also remember to complete the online pain score at daily bases. The team has experience of similar trials with the same group of participants, for which it has successfully recruited.

The findings from this pilot study will be disseminated to the scientific community, service users and policy**-**makers via submission to an Open Access peer**-**review journal. In addition the patient public involvement representatives are taking the lead to disseminate the findings to the service users.

## Trial status

The trial is still recruiting.

## Additional files

Additional file 1:
**CONSORT checklist.** (PDF 70 kb)
Additional file 2:
**Personal cover image 1: Study logo.** (DOC 21 kb)

## Abbreviations

APAP: auto**-**adjusting continuous positive airways pressure
CONSORT: Consolidated Standards of Reporting Trials
CPAP: continuous positive airways pressure
CRFs: Clinical Report Forms
HbSS: haemoglobin SS
LDH: lactate dehydrogenase
NIHR: National Institute of Health Research
PEDS**-**QL: Pediatric Quality of Life Inventory TM
POMS2a: Prevention of Morbidity in SCA pilot trial
POMS2b: Prevention of Morbidity in SCA Phase 2 trial
PPI: participants and public involvement
QOL: quality of life
RCT: randomised controlled trial
RfPB: Research for Participant Benefit
SCA: sickle cell anaemia
SpO_2_: oxygen saturation
WISC**-**^IV^UK: Wechsler Intelligence Scale for Children
