# Supplementary material for: Prevention of Morbidity in sickle cell disease - qualitative outcomes, pain and quality of life in a randomised cross-over pilot trial of overnight supplementary oxygen and auto-adjusting continuous positive airways pressure (POMS2a): study protocol for a randomised controlled trial
Source: Trials. 2015 Aug 25;16:376. doi: 10.1186/s13063-015-0883-y (PMC4548303; doi:10.1186/s13063-015-0883-y)
Supplement: Additional file 2: — Personal cover image 1: Study logo. (DOC 21 kb) [file 13063_2015_883_MOESM2_ESM.doc]

Additional file 2: Personal cover image 1: Study logo.
